# Keras Slim ResNet‐Based Prediction of Furcation Management Recommendations Among Dentists

**DOI:** 10.1155/ijod/6648299

**Published:** 2026-04-20

**Authors:** Pradeep Kumar Yadalam, Deepavalli Arumuganainar, Seyed Ali Mosaddad, Artak Heboyan, Navin Kumar Duraisami

**Affiliations:** ^1^ Department of Periodontics, Saveetha Dental College and Hospitals, Saveetha Institute of Medical and Technical Sciences, Saveetha University, Chennai, Tamil Nadu, India, saveetha.com; ^2^ Department of Conservative Dentistry and Prosthodontics, Department of Dentistry, Complutense University of Madrid, Madrid, Spain, ucm.es; ^3^ Department of Prosthodontics, Faculty of Stomatology, Yerevan State Medical University after Mkhitar Heratsi, Yerevan, Armenia, ysmu.am; ^4^ Department of Prosthodontics, School of Dentistry, Tehran University of Medical Sciences, North Karegar Street, Tehran, Iran, tums.ac.ir; ^5^ Department of Periodontics, Raja’s Dental College and Hospital, Chennai, Tamil Nadu, India

**Keywords:** dental, deep learning, furcation, machine learning, medical, periodontal disease

## Abstract

**Purpose:**

Furcation involvement occurs when periodontal deterioration reaches the roots of a multirooted tooth, making diagnosis and treatment difficult. Furcation management has shifted from periodontal care to unnecessary extraction and prosthetic replacement with or without implants. Prediction modeling with cutting‐edge machine learning algorithms was used to select treatments from questionnaire responses. We used Keras ResNet to forecast dentists’ furcation management recommendations.

**Materials and Methods:**

The participants were dentists with undergraduate or postgraduate degrees in fields other than periodontics and 5 years of practical experience. The study comprised 437 South Indian dentists aged 28–60 years. The author estimated the sample size based on previous research. We compared findings from a data robot tool employing a state‐of‐the‐art model with Keras Slim and Light Gradient Boosting. Data were split 80/20 between training and testing.

**Results:**

Keras ResNet and light gradient improved accuracy by 84% in predicting the target class of furcation‐related tooth treatment suggestions.

**Conclusion:**

The Keras Slim ResNet‐based questionnaire‐prediction model among dentists has demonstrated good accuracy, helping predict referral patterns for managing furcation‐involved teeth. In addition, it encourages general dentists to refer complex furcation cases to periodontists for expert care consistently.

## 1. Introduction

Furcation involvement is a pathological condition in which periodontal destruction extends to the area where the roots of a multirooted tooth divide, posing significant challenges for diagnosis and management [1, 2]. The primary challenge in diagnosing furcation involvement is its often asymptomatic nature in the early stages [3], making early detection difficult without routine periodontal assessments. Traditional methods like probing are used, but they are limited in accuracy, especially in posterior teeth, due to accessibility issues [4]. Moreover, the morphology of furcation areas varies significantly among individuals, complicating the identification of the exact extent of involvement. Radiographic evaluations are supplementary but can be misleading because these images are two‐dimensional and may not accurately represent the three‐dimensional structure of furcation areas [4].

Advanced diagnostic tools like cone‐beam computed tomography (CBCT) [1, 5] offer more detailed visualization, yet they are not routinely used due to higher costs and radiation exposure. Furthermore, it can be challenging to differentiate between furcation involvement caused by periodontal disease and that caused by other conditions, such as root caries or resorption.

Management of furcation involvement is multifaceted and depends on the severity of the condition. Nonsurgical methods, including scaling and root planing, are effective in the initial stages but have limited efficacy in advanced cases due to the inaccessibility of furcation areas [1]. Surgical interventions, such as open‐flap debridement, regenerative procedures, or resective surgeries, are often required for moderate‐to‐severe cases. However, these procedures are technically demanding and require careful consideration of the tooth’s strategic value [6, 7].

Patient compliance is another critical factor in managing furcation involvement. Long‐term success depends heavily on the patient’s adherence to oral hygiene practices and regular periodontal maintenance [7, 8]. Unfortunately, patient compliance can be variable, influenced by factors such as the complexity of home care instructions and individual patient motivation [4, 9, 10]. Thus, furcation involvement presents unique challenges in both diagnosis and management.

Decision‐making for furcation management has shifted from salvaging the tooth through periodontal management to unwarranted extraction, followed by prosthesis replacement, with or without implant support [4, 10, 11]. Hence, a questionnaire‐based survey was conducted among dental practitioners other than periodontists in South India to gather information on decision‐making regarding furcation‐involved teeth and their attitudes toward referring patients to periodontists for the management and preservation of the tooth and periodontium. The questionnaire results were subjected to predictive modeling using state‐of‐the‐art machine learning algorithms to assess their knowledge and attitudes regarding furcation involvement and their treatment recommendations. In predicting furcation management recommendations among dental practitioners, Keras Slim ResNet presents an innovative application of artificial intelligence (AI) technology. In this scenario, the predictive model is trained solely on data from a questionnaire‐based survey of dental clinicians, focusing exclusively on their insights and experiences, without using dental images.

This approach is distinct in its reliance on qualitative data gathered from dental practitioners. The questionnaire captures various variables, including dentists’ preferred treatment methodologies and attitudes toward patient referral to periodontists. By aggregating diverse professional knowledge, the survey provides a rich, multifaceted dataset that reflects the complexity of clinical decision‐making in furcation management.

Training the Keras Slim ResNet model on this dataset offers a unique perspective: the system learns to predict management strategies not from clinical images but from patterns and preferences inherent in expert human judgment. Therefore, this Keras Slim ResNet model reflects collective clinical wisdom, synthesizing a broad spectrum of professional experiences into its predictions. We aimed to identify and predict furcation management recommendations among dentists using Keras Slim ResNet.

## 2. Materials and Methods

This study was conducted in the Department of Periodontics, Ragas Dental College and Hospital, Chennai, India. The study was approved by the Institutional Ethics Committee (ECR/1163/Inst/TN/2018). Written Informed consent was obtained from all the participants. The participants were dental practitioners with undergraduate or postgraduate degrees other than a specialization in periodontics, and a minimum of 5 years of clinical experience. The study included 437 participants aged 28–60 years engaged in dental practice in South India. The sample size was estimated from the author’s previous study [9].

Participants were recruited via nonprobability convenience sampling through professional dental networks, institutional contacts, and electronic communication with South Indian dental practitioners. The online survey was voluntary. Of the 520 invited dentists, 437 completed the study, yielding an 84.0% response rate. Incomplete or ineligible responses were excluded before data analysis.

### 2.1. The Questionnaire and the Survey Method

A clinical scenario depicting a grade 2 furcation defect, accompanied by a comprehensive clinical narrative and radiographic depiction, was disseminated to participants via an online platform using a Google Form. The case demonstrated Glickman’s grade 2 classification, subclass B, with a 4 mm furcation defect in tooth 46, characterized by a probing pocket depth (PPD) of 6 mm and clinical attachment loss (CAL) of 7 mm. Intraoral periapical radiography unveiled vertical bone loss surrounding the distal root of tooth 46, extending into the apical third, coupled with inter‐radicular bone loss. The measurements of PPD and CAL were performed according to standard protocols. The patient’s medical, dental, and family history were unremarkable.

The questionnaire comprised 11 inquiries covering clinicians’ demographic details, professional attributes, and specific queries regarding the clinical case, including the nature of bone loss and suitable treatment modalities, as well as their inclination to refer patients to periodontists for tooth preservation and management. Refer to Table 1 for a detailed breakdown of the questionnaire along with corresponding normalization codes.

**Table 1:** The questionnaire and the corresponding responses with coding in numerals.

S. no	Questionnaire component	Responses and corresponding codes (numerals)
1	Age	1. <30 years 2. 31–40 years 3. 41–50 years 4. >50 years
2	Gender	1. Male 2. Female
3	Graduation level	1. BDS 2. MDS
4	Specialty practice	1. General practitioner 2. Endodontics 3. Pedodontics 4. Orthodontics 5. Prosthodontics 6. Oral medicine and radiology 7. Oral and maxillofacial pathology 8. Oral and maxillofacial surgery 9. Public health dentistry
5	Years in practice/consultation	1. <5 years 2. 5–10 years 3. 10–20 years 4. >20 years
6	What do you observe in this case?	1. Only interdental bone loss 2. Only inter‐radicular bone loss 3. Endo‐perio lesion 4. a + b 5. All the above
7	What line of treatment would you recommend for this tooth?	1. Only endodontic management 2. Only periodontal management 3. a b 4. Extraction and fixed prosthesis 5. Extraction and implants
8	Why do you suggest this treatment plan?	1. It seems to be appropriate for this clinical scenario 2. Not sure if periodontal management is required 3. Usually, periodontal management is unpredictable 4. More confident with this treatment modality
9	Where do you obtain information on periodontal disease and its management?	1. Only specialty journals Only specialty journals 2. Only CDE programs 3. Only the internet 4. a + b 5. b + c 6. All the above 7. Others
10	Have you attended CDE program lectures on periodontal disease and management?	1. Yes, by periodontists 2. Yes, by dental practitioners other than periodontists 3. a + b 4. Never
11	How often do you refer/consult a periodontist for periodontal management?	1. Always 2. Occasionally 3. Never

The authors initially created the questionnaire in English and conducted a pilot survey with 30 participants to validate it. Internal consistency was strong, with a Cronbach’s *α* of 0.82. Face validity was assessed following Lawshe’s [12] 1975 method, involving a pretest group of experienced periodontists. Consequently, the same questionnaire used in the pilot study was also used in the main study. The examiner distributed the questionnaire along with case descriptions to each participant, allowing up to 30 min for completion.

### 2.2. Dataset Preparation

Data was retrieved from this questionnaire; outliers were removed and normalized for consistency. The target variable was treatment recommendation, and the dataset was divided into a training set and a test set. Model development and validation were performed using the DataRobot platform on clinician‐reported questionnaire responses. Automated preprocessing, feature engineering, model benchmarking, hyperparameter tuning, and stratified cross‐validation were conducted within the platform. A cross‐validation technique was employed to enhance model robustness. Each fold served as both a training (80%) and a test set (20%), reducing the risk of overfitting. This methodology facilitated the training and evaluation of predictive models for determining treatment recommendations in dental practice. Using the data robot tool, this data was analyzed for Keras Slim neural networks and light gradient boost trees.

The target variable was derived from Question 7, where clinicians recommended treatment options: 1 = endodontic only, 2 = periodontal only, 3 = combined, 4 = extraction with prosthesis, and 5 = extraction with implant. These were encoded as categories, framing the task as a multiclass classification. During training, labels served as outcomes, and other responses as features. Performance was assessed via accuracy and AUC, using a one‐vs‐rest approach in the modeling pipeline. Although the questionnaire included a response category of “<5 years of clinical experience”, the study inclusion criteria required participants to have at least 5 years of clinical practice. Therefore, responses from participants with less than 5 years of experience were excluded during data preprocessing before model development.

### 2.3. Keras Slim Residual Network

Keras Slim Residual Network, also known as ResNet, is a deep learning architecture that enables the effective training of very deep neural networks. It is designed to address the vanishing gradient problem, which occurs when gradients become increasingly small as they propagate through multiple layers, making it difficult for the network to learn effectively. ResNet introduces the concept of residual blocks, which allow the network to learn residual functions. The basic building block of a residual network is the identity shortcut connection, which skips one or more layers. This shortcut connection allows gradients to flow more easily through the network, thereby improving training. The architecture of a ResNet typically includes several stacks of residual blocks, followed by a global average pooling layer and a fully connected layer for classification. The residual blocks can have different depths and configurations depending on the ResNet variant (ResNet‐50, ResNet‐101, etc.).

The Keras Slim Residual Network (ResNet) architecture is a deep learning model that mitigates the challenges of training very deep neural networks effectively. ResNet achieves this by introducing residual blocks, which enable the network to learn residual functions. Each residual block consists of convolutional layers with batch normalization and activation functions, along with a shortcut connection that bypasses one or more layers. This shortcut connection allows gradients to flow more smoothly, addressing the vanishing‐gradient problem. ResNet has multiple residual blocks, followed by global average pooling and a fully connected layer for classification. Its configuration varies by ResNet version, like ResNet‐50 or ResNet‐101. ResNet is a powerful architecture that excels in image classification, object detection, and semantic segmentation.

The pipeline tested multiple algorithms using a fivefold stratified cross‐validation. The chosen Keras Slim Residual Network had residual blocks, batch normalization, ReLU, global pooling, and a multiclass output. Parameters included learning rate, adaptive batch normalization, and regularization tuning. For light gradient boosting, parameters such as estimators, learning rate, maximum depth, and feature subsampling were automatically optimized to improve performance.

Model building used DataRobot’s AutoML platform, where the dataset was uploaded, and feature preprocessing, algorithm selection, hyperparameter tuning, and ensembling were automated. The model was assessed via stratified fivefold cross‐validation. The best model was a gradient‐boosted tree ensemble, with parameters such as tree depth, learning rate, estimators, and subsampling automatically tuned to maximize AUC. The final metrics were computed on a validation set not used in training.

### 2.4. Light Gradient Boosts Trees

The light gradient boosted tree (LightGBM) is a machine learning algorithm that belongs to the family of gradient boosting methods. It is designed to handle large‐scale, high‐dimensional datasets efficiently and has gained popularity for its speed and accuracy. The architecture of LightGBM consists of multiple decision trees trained sequentially. Each tree is built greedily, where at each step the algorithm selects the best split based on a specific criterion (usually the reduction in the loss function, e.g., the mean squared error for regression or log loss for classification). Unlike traditional gradient boosting methods, LightGBM employs two major techniques to improve performance: Gradient‐based one‐side sampling (GOSS) and exclusive feature bundling (EFB). GOSS is a data sampling technique that retains a subset of training instances with large gradients while randomly sampling the rest. This allows LightGBM to focus on critical data points and generate trees with better generalization, making it faster and more accurate.

LightGBMs is an ensemble method that sequentially constructs multiple decision trees. It uses GOSS and EFB techniques to improve generalization ability and training speed. By combining these techniques with the gradient boosting framework, LightGBM provides a scalable solution for handling large datasets.

## 3. Results

Among 436 clinicians, the most common recommendation was combined endodontic–periodontal management (*n* = 211; 48.4%), followed by extraction with implant placement (*n* = 121; 27.8%). Only periodontal management was selected by 51 (11.7%), extraction with fixed prosthesis by 41 (9.4%), and only endodontic management by 12 (2.8%).

### 3.1. Lift Chart Assessment

**Figure 1:**
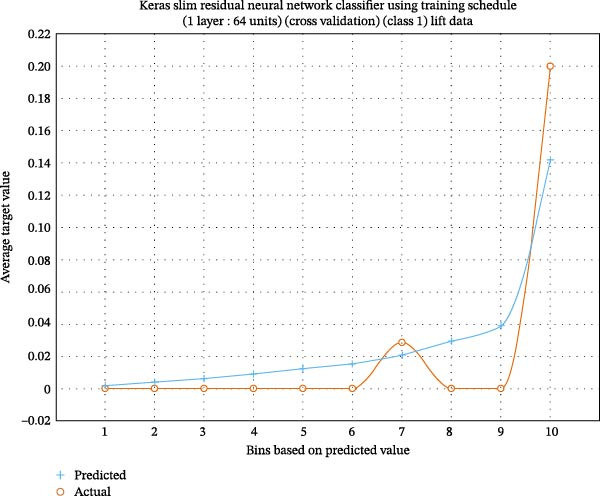
Lift chart of Keras’s slim residual network of the predicted class, with high lift showing good accuracy.

**Figure 2:**
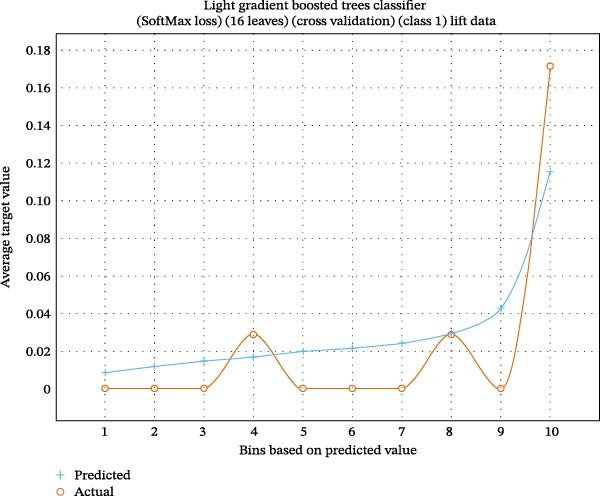
Lift chart of the light gradient boosted tree for the predicted class, with high lift indicating accuracy for that class.

Lift charts are used to compare prediction models against baselines, displaying the cumulative proportion of the target variable as a function of population ranked by estimated likelihood. They demonstrate how well a model ranks individuals as more likely to undertake a specific activity than expected by chance (Figures 1 and 2).

### 3.2. Confusion Matrix

**Figure 3:**
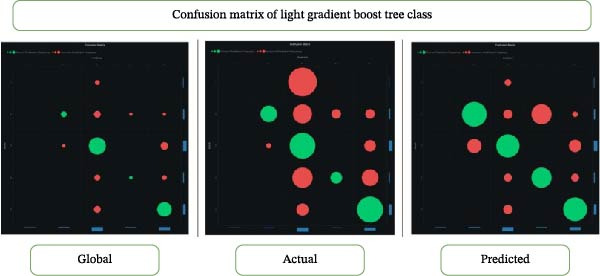
Confusion matrix of the light gradient boosted tree class.

**Figure 4:**
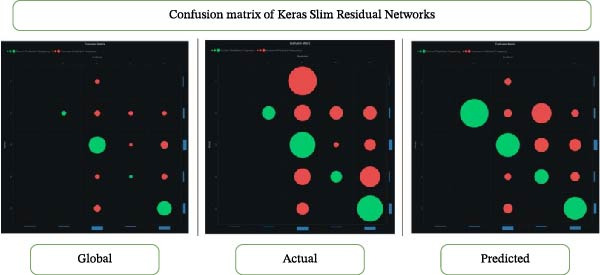
Confusion matrix of the Keras Slim Residual Network.

Machine learning and statistics extensively employ confusion matrices. The confusion matrix evaluates a classification model’s performance by showing its accuracy and the number of incorrect predictions on a test dataset. It represents four possible outcomes: true positives (TP), false positives (FP), true negatives (TN), and false negatives (FN). The matrix provides a comprehensive view of the model’s performance, allowing comparisons across classes and determining whether it favors one class. Performance metrics often use the F1 score (Figures 3 and 4).

Table 2 shows the comparison of Keras ResNet and light gradient boosted, with an accuracy of 84%, and both models performed equally in predicting the target class of recommendations for furcation.

**Table 2:** The comparison between Keras ResNet and light gradient regarding AUC and LOG LOSS.

S. no	AUC (%)	LOG LOSS
Keras slim residual neural network	84	0.9161
Light gradient boost tree classifier	84	0.8950

Both the Keras slim residual neural network and the light gradient boosting tree classifier achieved an AUC of 84% for treatment recommendation prediction. However, the light gradient boosting tree classifier outperformed the Keras Slim ResNet on the log loss metric, suggesting it may be more accurate.

## 4. Discussion

The management of furcation involvement poses a significant challenge due to its inherent complexity and critical importance [13]. Effectively addressing furcation problems requires precision and expertise, as neglecting them can lead to tooth loss and complications. Dentists must navigate intricate treatment options to salvage the tooth and preserve its function and esthetics, using techniques such as guided tissue regeneration or appropriate osseous surgery. The complexity lies in accurately diagnosing the condition and opting for the appropriate intervention to achieve successful outcomes [11]. Despite its complexity, furcation management is vital for maintaining oral health and patient well‐being. The findings should be viewed as decision‐support, not autonomous decision‐making. The machine‐learning model aims to assist clinicians by recognizing response patterns and aiding referral decisions, not replacing professional judgment in periodontal diagnosis and treatment planning.

Several factors, such as age, gender, years of practice, degree of specialization, year of graduation, number of observations, treatment plan recommendations, information source, attendance at CDE program lectures, and consultation with periodontists, can affect the treatment recommendation for furcation involvement. Because younger practitioners may have less experience with the outcomes of furcation management, age may influence the recommended treatment plan. The recommended course of therapy may not be directly impacted by gender. Graduation level may also affect the recommended course of care, as dentists with greater training and experience may be better equipped to manage furcation involvement. The recommendation for treatment may also be influenced by the dentist’s years of experience, as they may have handled cases similar to yours and have a better understanding of therapy outcomes. A specific treatment plan may be recommended based on factors such as clinical appropriateness, confidence in the treatment modality, and predictability of periodontal management.

Dentists sometimes hesitate or overlook the need for specialized care in furcation cases, leading to suboptimal outcomes [14]. The Keras Slim ResNet–based model may aid decision‐making by identifying response patterns and indicating when specialist referral is needed [15]. This ensures better patient outcomes and the overall quality of dental healthcare by leveraging technology to optimize referrals, a crucial aspect of dental practice. The Keras Slim ResNet–Based Questionnaire Prediction model is a pivotal advancement in dentistry, bridging the gap between general and specialized care to benefit patients and the dental profession [15]. A previous study examined general dentists’ views on the involvement of periodontal furcation (FI). Online surveys of GDPs in seven nations yielded 400 replies [9]. Some practitioners did not utilize six‐point pocket charts, and a few felt safe treating FI. Country and qualifying year affected replies. Insufficient expertise and equipment hindered FI management.

In a clinical setting, the proposed Keras Slim ResNet–based model acts as a decision‐support tool rather than replacing professionals. When a general dentist encounters a tooth with furcation involvement, they can enter relevant clinical and practitioner‐related data, similar to the data in the questionnaire, into the system. The model then predicts management options based on patterns of expert decision‐making. This allows clinicians to compare their approach with evidence‐based predictions, supporting more consistent and rational treatment planning. In practice, it can serve as a second‐level advisory tool, especially when prognosis is uncertain or referral is needed. It also serves as an educational aid for early career dentists and those with limited experience in complex cases. By reinforcing referral patterns aligned with current principles, it promotes learning, awareness of furcation management, and timely specialist consultation. This can lead to more standardized decisions, increased confidence for less experienced clinicians, and better patient outcomes. The proposed model serves as a probabilistic decision‐support system that complements clinical judgment by aggregating clinician‐reported data to inform referral decisions. All predictions depend on questionnaire design, data, and algorithms, and, like all machine‐learning tools, are subject to bias and should be interpreted cautiously within clinical workflows.

The study proposes improving general dentists’ FI management skills, as this can affect public health [16]. Another study found that most Victorian dentists are confident in diagnosing and treating typical cases of periodontal disease but less confident in detecting severe or aggressive cases. An early study surveyed 355 Northern Ireland general dentists [17, 18] and found that practice location, dissatisfaction with National Health Service treatment, past referral refusals, poor postgraduate education, and nondisease criteria, such as specialized service availability, significantly influence referral decisions.

In another study, general dentists recommended periodontal therapy to their patients, with 90.4% trusting its efficacy [19, 20]. General dentists conducted most periodontal operations and phase I treatment, but referred cases to specialists for grafting, ridge augmentation, and dental implants. The authors proposed additional continuing dental education programs to modify general dentists’ referral patterns for periodontal therapy and to enhance their awareness of disease progression and treatment outcomes. Previous literature [21–23] did not examine predictive modeling of questionnaire‐based periodontal treatment referral for furcation. Therefore, we used machine learning to gain deeper insights and to help periodontists understand why dentists are unaware of current developments in furcation management. Questionnaire‐based machine learning research evaluates survey data using machine learning techniques. These studies anticipate outcomes or find patterns in questionnaire replies. Our investigation found that Keras ResNet and light gradient boosting had 84% accuracy in predicting “furcation suggestions” (Figures 2–4; Table 2).

Machine learning‐based questionnaire‐based mortality studies were developed using machine learning [24, 25]. and Cox regression on two Chinese populations [26]. The model best predicted 6‐year mortality, with a C‐index of 0.86. Fuzzy Forests analysis revealed significant variation in health outcomes between non‐English and English responses compared to our Keras Slim ResNet, and light gradient boost showed good accuracy of 84% in predicting treatment outcome in furcation management.

Although the model showed promising predictive performance, the dataset’s composition should be considered when interpreting the findings. Responses were from dental practitioners in a specific region and clinical environment. While the dataset represents various specialties and experience levels, it may not reflect practice differences across institutions, regions, or healthcare systems. Thus, the model’s generalizability to broader or international populations may be limited. Future multicenter and geographically diverse studies are needed to improve external validity and clinical applicability.

This study suggests future directions for neural networks and gradient boosting models in forest management. These include expanding the dataset, incorporating additional features, fine‐tuning hyperparameters, validating external datasets, and improving model interpretability and explainability. However, limitations include data availability and quality, generalizability, treatment recommendations, and interpretation of complex predictions. The models’ accuracy may not be universally applicable across diverse settings, and they may require frequent updates to remain relevant. The model is a clinical decision‐support tool aiding practitioners in evaluating management options. Final treatment decisions should still be based on clinical examination, practitioner expertise, and individual patient needs factors. Additionally, the models’ black‐box nature may limit trust in clinical decision‐making. Ethical considerations include ensuring models do not introduce bias or discrimination, and regular monitoring and evaluation to prevent bias or disparities in predictions.

## 5. Conclusion

This study shows that an automated machine learning–based questionnaire‐prediction model can identify patterns in clinicians’ furcation management recommendations, with moderate predictive performance. It highlights variability among nonperiodontist dental practitioners and the potential of probabilistic decision‐support tools to inform understanding of referral behavior. Though exploratory, these results lay the foundation for future studies to develop clinically useful referral‐support systems in periodontal care. The Keras Slim ResNet model streamlines referrals for furcation‐involved teeth by using algorithms and patient data to assess the need for specialized periodontal care. This system improves patient care by identifying candidates and prioritizing access to specialists. More research is needed to fully validate and enhance the model’s predictive capabilities and assist general dentists in recommending patients to periodontists.

## Author Contributions

Conceptualization, methodology, software, formal analysis, investigation, data curation, writing – original draft preparation: Pradeep Kumar Yadalam and Deepavalli Arumuganainar. Validation, supervision, project administration: Pradeep Kumar Yadalam and Artak Heboyan. Resources: Seyed Ali Mosaddad and Artak Heboyan. Writing – review and editing: Seyed Ali Mosaddad, Navin Kumar Duraisami, and Artak Heboyan. Visualization: Seyed Ali Mosaddad.

## Funding

The authors have nothing to report.

## Disclosure

All authors have read and approved the published version of the manuscript.

## Ethics Statement

This study was conducted in accordance with the Declaration of Helsinki (2013). Ethical approval was obtained from the Institutional Human Ethics Committee of Ragas Dental College and Hospital, Chennai, India (Approval Number ECR/1163/Inst/TN/2018).

## Consent

All participants were informed about the study objectives, and written informed consent was obtained prior to participation.

## Conflicts of Interest

The authors declare no conflicts of interest.

## Data Availability

The data presented in this study are available upon request from the corresponding author. Due to ethical approval restrictions related to human participant questionnaire data and the proprietary nature of the DataRobot platform, individual‐level datasets and pretrained model weights cannot be publicly released. Aggregated anonymized outputs and methodological details are available from the corresponding author upon reasonable academic request.

## References

[1] Das R. K. , Bharathwaj V. V. , and Sindhu R. , et al.Comparative Analysis of Various Forms of Local Drug Delivery Systems on a Class 2 Furcation – A Systematic Review, Journal of Pharmacy and Bioallied Sciences. (2023) 15, no. Suppl 1, S742–S746, 10.4103/jpbs.jpbs_572_22. 37654351 PMC10466541

[2] Trullenque-Eriksson A. , Tomasi C. , Petzold M. , Berglundh T. , and Derks J. , Furcation Involvement and Tooth Loss: A Registry-Based Retrospective Cohort Study, Journal of Clinical Periodontology. (2023) 50, no. 3, 339–347, 10.1111/jcpe.13754. 36415171

[3] Lee B. L. , Soukup J. , Rendahl A. , and Goldschmidt S. , Clinical Success of Guided Tissue Regeneration for Treating Vertical Bone and Furcation Defects in Dogs, Frontiers in Veterinary Science. (2023) 10, 10.3389/fvets.2023.1247347, 1247347. 37711437 PMC10498771

[4] Limiroli E. , Calò A. , Limiroli A. , Cortinovis I. , and Rasperini G. , Radiographic Ratios for Classifying Furcation Anatomy: Proposal of a New Evaluation Method and an Intra-Rater and Inter-Rater Operator Reliability Study, Clinical Oral Investigations. (2023) 27, no. 4, 1541–1546, 10.1007/s00784-022-04774-6. 36781478 PMC10102072

[5] Chatzopoulos G. S. , Koidou V. P. , and Tsalikis L. , Local Drug Delivery in the Treatment of Furcation Defects in Periodontitis: A Systematic Review, 2023, 27, 955–970, Clinical Oral Investigations. 10.1007/s00784-023-04871-0 PMC9985576 36729235

[6] Nibali L. , Shemie M. , and Li G. , et al.Periodontal Furcation Lesions: A Survey of Diagnosis and Management by General Dental Practitioners, Journal of Clinical Periodontology. (2021) 48, no. 11, 1441–1448, 10.1111/jcpe.13543. 34472119

[7] Hicks M. J. , Uldricks J. M. , Whitacre H. L. , Anderson J. , and Moeschberger M. L. , A National Study of Periodontal Assessment by Dental Hygienists, Journal of Dental Hygiene: JDH/American Dental Hygienists’ Association. (1993) 67, no. 2, 82–92. 17233170

[8] Ittycheria P. G. , Veliyaveetil T. G. , George A. K. , John S. , Thomas N. G. , and Cherian S. A. , Effectiveness of Platelet-Rich Fibrin With Decalcified Freeze-Dried Bone Allograft Compared to Decalcified Freeze-Dried Bone Allograft Alone in Mandibular Grade–II Furcation Defects: A Quasi-Experimental Study, Pesquisa Brasileira em Odontopediatria e Clínica Integrada. (2023) 23, 10.1590/pboci.2023.049, e210126.

[9] Arumuganainar D. , Arun K. V. , Alamelu S. , Elango S. S. , Pitchumani P. K. , and Ponnusamy K. , Referral Patterns for Furcation Management Among Dental Clinicians in an Academic Setting—A Preliminary Study, Journal of Clinical and Diagnostic Research. (2022) 16, no. 1, ZC30–ZC37, 10.7860/JCDR/2022/45446.15904.

[10] Limiroli E. , Calò A. , and Cortellini P. , et al.The Influence of Interradicular Anatomy on the Predictability of Periodontal Regenerative Therapy of Furcation Defects: A Retrospective, Multicenter Clinical Study, Clinical Oral Investigations. (2023) 27, no. 7, 3779–3786, 10.1007/s00784-023-04995-3. 37052671 PMC10329584

[11] Garg N. , Lamba A. K. , Faraz F. , Tandon S. , Datta A. , and Dhingra S. , Management of Grade II and III Furcation Defects With Intramarrow Penetration Along With Indigenously Prepared DFDBA and Amniotic Membrane: A Clinical and Radiographic Study, Cell and Tissue Banking. (2024) 25, no. 1, 295–303, 10.1007/s10561-022-10068-8. 36627541

[12] Lawshe C. H. , A Quantitative Approach to Content Validity, Personnel Psychology. (1975) 28, no. 4, 563–575.

[13] Shaikh M. S. , Fareed M. A. , and Zafar M. S. , Bioactive Glass Applications in Different Periodontal Lesions: A Narrative Review, Coatings. (2023) 13, no. 4, 716.

[14] Anamika , Diagnosis, A Predominant Aspect in Furcation Management: A Review, Acta Scientific Dental Scienecs. (2023) 7, no. 4, 14–18.

[15] Dy K. , Ligan J. , and Cabatuan M. , Understanding and Coding a ResNet in Keras, Journal of Telecommunication, Electronic and Computer Engineering. (2018) 10.

[16] Menhadji P. , Patel R. , and Asimakopoulou K. , et al.Patients’ and Dentists’ Perceptions of Tele-Dentistry at the Time of COVID-19. A Questionnaire-Based Study, Journal of Dentistry. (2021) 113, 10.1016/j.jdent.2021.103782, 103782. 34400252 PMC8361006

[17] Taheri M. H. , Eshraqi A. M. , Anwari A. , and Stanikzai A. M. , Prevalence of Recurrent Aphthous Ulcers Among Dentistry Students’ in Kabul, Afghanistan: A Questionnaire-Based Study, Clinical, Cosmetic and Investigational Dentistry. (2022) 14, 275–279, 10.2147/CCIDE.S378171. 36132195 PMC9482951

[18] Imorde L. , Möltner A. , Runschke M. , Weberschock T. , Rüttermann S. , and Gerhardt-Szép S. , Adaptation and Validation of the Berlin Questionnaire of Competence in Evidence-Based Dentistry for Dental Students: A Pilot Study, BMC Medical Education. (2020) 20, no. 1, 10.1186/s12909-020-02053-0, 136. 32366287 PMC7197120

[19] Salajegheh M. , Hekmat S. N. , and Malekpour-afshar R. , Identification of Alternative Topics to Diversify Medicine, Dentistry, and Pharmacy Student Theses: A Mixed Method Study, BMC Medical Education. (2023) 23, no. 1, 10.1186/s12909-023-04031-8, 110. 36782213 PMC9923902

[20] Karaman A. and Sapan Z. , Evaluation of Temporomandibular Disorders, Quality of Life, and Oral Habits Among Dentistry Students, CRANIO®. (2023) 41, no. 4, 316–322, 10.1080/08869634.2020.1857615. 33325334

[21] Acharya A. , Chodankar R. N. , Patil R. , and Patil A. G. , Assessment of Knowledge, Awareness, and Practices Toward the use of 3D Printing Among Dental Laboratory Technicians in Karnataka, India: A Cross-Sectional Study, Journal of Oral Biology and Craniofacial Research. (2023) 13, no. 4, 476–481, 10.1016/j.jobcr.2023.05.006. 37250816 PMC10220251

[22] Khan H. A. , Mathur A. , Nair S. M. , and Shetty S. , Knowledge and Attitudes of Parents Regarding Digit Sucking Habit in Children in Pune: A Questionnaire-Based Cross-Sectional Study, Journal of Clinical and Diagonostic Research. (2023) 17, no. 7, SC06–SC10, 10.7860/JCDR/2023/62371.18145.

[23] Lee J. N. , Hill C. M. , and Chi D. L. , Using Policy Briefs to Communicate Dental Research Findings to Policymakers, JDR Clinical and Translational Research. (2024) 9, no. 2, 150–159, 10.1177/23800844231171831. 37317831

[24] Kumar V. S. , Kumar P. R. , and Yadalam P. K. , et al.Machine Learning in the Detection of Dental Cyst, Tumor, and Abscess Lesions, 2023, Research Square. 10.1186/s12903-023-03571-1 PMC10626702 37932703

[25] Yadalam P. K. , Trivedi S. S. , and Krishnamurthi I. , et al.Machine Learning Predicts Patient Tangible Outcomes After Dental Implant Surgery, IEEE Access. (2022) 10, 131481–131488, 10.1109/ACCESS.2022.3228793.

[26] Li Z. , Yang N. , and He L. , et al.Development and Validation of Questionnaire-Based Machine Learning Models for Predicting All-Cause Mortality in a Representative Population of China, Frontiers in Public Health. (2023) 11, 10.3389/fpubh.2023.1033070, 1033070. 36778549 PMC9911458

